# Current Role and Future Frontiers of Spatial Transcriptomics in Genitourinary Cancers

**DOI:** 10.3390/cancers17172774

**Published:** 2025-08-26

**Authors:** Firas Hatoum, Adnan Fazili, Justin W. Miller, Xuefeng Wang, Xiaoqing Yu, Xin Lu, Jeffrey S. Johnson, Philippe E. Spiess, Jad Chahoud

**Affiliations:** 1 Department of Genitourinary Oncology, H. Lee Moffitt Cancer Center and Research Institute, Tampa, FL 33612, USA; firas.hatoum@moffitt.org (F.H.); adnan.fazili@moffitt.org (A.F.); jeffrey.johnson@moffitt.org (J.S.J.); 2 USF Health Morsani College of Medicine, Tampa, FL 33602, USA; 3 Department of Biostatistics and Bioinformatics, H. Lee Moffitt Cancer Center and Research Institute, Tampa, FL 33612, USA; xuefeng.wang@moffitt.org (X.W.); xiaoqing.yu@moffitt.org (X.Y.); 4 Department of Biological Sciences, University of Notre Dame, Notre Dame, IN 46556, USA; xlu@nd.edu

**Keywords:** spatial transcriptomics, genitourinary oncology, tumor microenvironment, spatial gene expression, immunotherapy biomarkers, precision medicine

## Abstract

Malignant genitourinary tumors have demonstrated a rising incidence and are characterized by significant morbidity and mortality. Advances in sequencing technologies have greatly enhanced our understanding of the genetic and molecular mechanisms that drive tumor biology. In recent years, spatial transcriptomics technologies have rapidly evolved, enabling precise quantification and visualization of gene expression within the spatial context of tissues. Unlike conventional transcriptomics, which focuses on gene expression levels, spatial transcriptomics provides additional insights into the spatial distribution of genes within tissues, cellular composition, and cell-to-cell interactions within biological samples. In this narrative review, we outline the emerging role and future potential of spatial transcriptomics technology in genitourinary cancers.

## Simple Summary

Spatial transcriptomics is an advanced technology that allows for the study of gene activity in cancer tissues while preserving the original tissue structure. Unlike traditional methods that either analyze samples without location information or examine individual cells removed from their natural environment, spatial transcriptomic technology maps where genes are active within intact tumor tissue. This approach enables a greater understanding of how different areas of a tumor vary, how cancer cells interact with surrounding healthy tissue, and how cells are organized spatially within the tumor at high resolution. As spatial technology becomes more widely used, it may improve cancer diagnosis and treatment by helping to create more personalized and effective therapies for individual patients. This review outlines the current and future role of spatial transcriptomics methodologies in the context of genitourinary cancers.

## 1. Introduction

Cancer is a complex and heterogeneous disease [1]. Genitourinary (GU) malignancies including prostate, urothelial, kidney, penile, and testicular cancers are no exception, and collectively cause significant morbidity and mortality globally accounting for over 1 in 8 of all cancer diagnoses worldwide which results in nearly 2.6 million new cases and 795,000 deaths annually [2]. Carcinogenesis and disease progression can be characterized by intricate interactions between tumor cells and their surrounding microenvironment [3].

The origins of spatial transcriptomics (ST) can be traced to the late 20th century, when techniques like radioactive in situ hybridization (ISH) and chromogenic ISH allowed for ISH of mRNA within tissue sections. These approaches provided information about the location of specific mRNA but were limited in their ability to analyze many genes at once [4]. True multiplexing was achieved using fluorescently labeled probes upon the introduction of fluorescence in situ hybridization (FISH) in the 1990s, which paved the way for much more sophisticated approaches in ST [4].

Microdissection-based approaches such as laser capture microdissection (LCM), which allow the isolation of specific tissue regions for downstream RNA sequencing, represented a significant advancement in the field; however, were labor-intensive and removed true spatial continuity from specimens [4]. The deficiencies in LCM fomented the development of more automated, high-throughput methods [4].

During the 2010s, spatial barcoding techniques represented a significant technological advancement [4]. One of the first widely recognized STs methods was pioneered by the Lundeberg group at SciLifeLab in Sweden, where barcoded oligonucleotides were printed on glass slides to capture mRNA from tissue sections while preserving spatial organization [4]. This technology laid the foundation for commercial platforms such as 10x Genomics Visium, one of the most predominant ST tools today [4].

In parallel with advances in spatial barcoding technology, single-molecule imaging techniques began to proliferate [4]. Among the various approaches, multiplexed error-robust FISH and sequential fluorescence in situ hybridization (seqFISH) utilized combinatorial labeling and sequential hybridization, respectively, advancing transcriptome-wide spatial profiling at single-cell resolution. Each methodology resolved gene throughput limitations of their earlier FISH-based ancestors and enabled high-dimensional spatial mapping [4].

More recently, spatial proteomics and multi-omics integration have expanded sequencing capabilities beyond RNA expression. Platforms such as NanoString CosMx, Akoya’s CODEX, and Spatial Molecular Imaging (SMI) now enable simultaneous spatial profiling of RNA and proteins at subcellular resolution, further enriching our understanding of tissue architecture and cellular interactions [4].

Advances in ST have significantly advanced our understanding of tissue biology, with the ability to profile gene expression in its spatial context. Unlike traditional bulk or single-cell RNA sequencing (scRNA-seq), which does not provide spatial resolution, techniques of ST can map transcriptional activity within intact tissues, which enables insights into cellular interactions and microenvironment dynamics [5]. Importantly, this capability is crucial in oncology, because tumor heterogeneity and immune microenvironment composition serve as critical components of oncologic disease progression and response to treatment [6].

ST shows promise for enhancing diagnostic, prognostic, and therapeutic approaches in genitourinary cancers. Bladder, kidney, and prostate carcinomas show high spatial heterogeneity, which in turn affects their treatment resistance and potentiates immune evasion [7]. ST technology allows for investigation of the tumor-immune interaction, identification of niche-specific biomarkers, and spatial dynamics of treatment response [8]. Integration of ST data with other omics, including spatial proteomics and metabolomics, could contribute to a comprehensive molecular atlas of GU malignancies to be used toward precision oncology strategies [8].

Despite significant progress in the characterization of these malignancies, several challenges remain, including deconvoluting intra-tumoral heterogeneity, determining mechanisms of therapeutic resistance, identifying predictive biomarkers, and understanding discrepancies in treatment response [9]. This narrative review synthesizes current knowledge and emerging applications of spatial transcriptomics in genitourinary cancers, evaluates its impact on our understanding of GU tumor biology, and assesses the translational potential of this technology for advancing clinical practice.

## 2. Spatial Transcriptomics Technology and Considerations for Genitourinary Oncology

### 2.1. Evolution of Transcriptional Profiling

**Figure 1:**
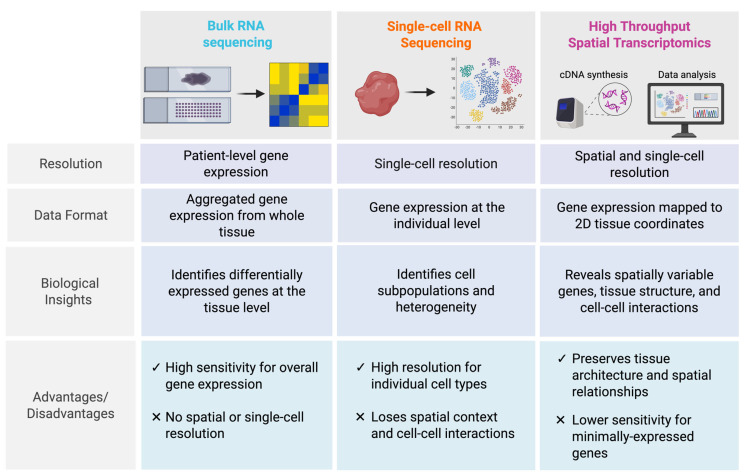
Comparison of bulk RNA-Seq, single-cell RNA-Seq, and high-throughput spatial transcriptomics.

Prior to the utilization of ST, bulk and scRNA-seq were the predominant methods for investigating gene expression at the molecular level. Bulk RNA sequencing involves homogenization of tissue samples and extraction of RNA during processing and enables analysis of gene expression in aggregate from an entire specimen [9]. ScRNA-seq allows for gene expression analysis at high-resolutions but similarly requires the dissociation of tissues during sample processing, which eliminates the spatial context of tumor specimens [5] (Figure 1).

The transition to the current era of ST has been driven by improvements in computational power and advancements in technologies such as next-generation sequencing (NGS) [10].

ST technology is distinct from traditional RNA sequencing techniques in that tissue architecture is preserved, thus allowing for gene expression analysis with additional insights into the spatial localization and interaction of cells [11]. This integration of spatial and molecular information provides critical insights into tumor heterogeneity, microenvironment interactions, and the complex cellular networks that drive cancer biology. Preserving the spatial context of cellular interactions is of importance in GU cancers due to their complex architectural features [12], distinct patterns of invasion [13], and heterogeneous immune infiltration within tumor immune microenvironment [14]. Spatial transcriptomics may be particularly valuable in the realm of genitourinary cancers, where extensive tumor heterogeneity and incomplete understanding of disease progression have historically limited advances in screening, risk stratification, and drug development.

Development of ISH began in the 1970s where early methods utilized radiolabeled probes to localize specific RNA within cells [15]. FISH techniques have since evolved and now enable quantitative RNA localization within single cells at subcellular resolution [16]. Further refinement in image-based ST methods, such as seqFISH, which utilizes multiple rounds of ISH to elucidate sequence information and spatial location, has allowed simultaneous detection of hundreds of RNA molecules [16]. Methods such as LCM can achieve high resolution, but are constrained by low throughput and technical challenges [17]. Collectively, these techniques can map the locations of several hundred to several thousand genes within tissue samples, rather than provide a comprehensive transcriptomic profile.

A variety of ST platforms are currently commercially available, and ongoing efforts seek to improve cost, resolution, acquisition times, and data analysis techniques [17]. Each system employs a unique strategy to capture and analyze spatial gene expression data, balancing factors such as resolution, sample throughput, and tissue compatibility, with several overarching methodological categories including sequencing-based (e.g., Xenium, Visium, Stereo-seq, and Seeker), hybridization-based (e.g., CosMx and MERSCOPE), cyclic imaging (e.g., PhenoCycler, COMET, CellScape), UV-based (GeoMx), and mass spectrometry (MIBIscope) transcriptomics [17,18,19] (Table 1).

### 2.2. Platform Selection and Considerations for GU Cancer Applications

As spatial technologies continue to evolve, selecting the appropriate platform for GU cancer applications requires careful consideration of available sample types, batch processing capabilities, resolution requirements, time constraints, and cost and infrastructure limitations [18,19,20]. Optimizing platform selection depends not only on the biological question being addressed, but also on these practical constraints and implementation requirements [18,19,20].

#### 2.2.1. Spatial Transcriptomic Sample Types

Sample type considerations represent a critical factor in platform selection. Most ST platforms can accommodate formalin-fixed paraffin-embedded (FFPE) or fresh-frozen tissue; however, some platforms are restricted to analysis of only a single sample type. Additionally, minimum tissue requirements vary across platforms, ranging from whole tissue sections to sub-millimeter regions [20]. As a result, the availability and quantity of fresh and preserved tissue limit the types of analysis that are possible [20]. FFPE tissue enables retrospective analyses of archived specimens and may help facilitate clinical biomarker validation studies [20]. In contrast, fresh-frozen tissue samples provide superior RNA quality and allow for improved molecular resolution; however, the samples must either be collected prospectively or retrieved from biorepositories with specialized storage infrastructure, thus limiting the ability to perform retrospective analyses on historical cohorts [20].

Moreover, GU cancers present tissue-specific challenges in the context of ST analyses [21]. For example, prostate tumor tissue is highly heterogenous, containing normal cells, tumor cells, and stromal cells in one tissue section, which complicates the interpretation of results [21]. Similarly, bladder cancer tissues are often difficult to preserve due to their high-water content and fragile nature, which significantly deteriorates RNA quality [22]. These challenges necessitate further methodological optimizations of protocols in terms of processing and preserving tissue from GU cancers.

#### 2.2.2. Spatial Transcriptomic Resolution Requirements

Another limitation of ST technologies is their spatial resolution, which determines the precision of gene expression mapping [23]. Many of the currently available technologies capture gene expression over regions containing multiple cells, leading to signal contamination and difficulties in distinguishing individual cell types [24]. For example, technologies like 10X Genomics Visium, with a resolution of approximately 50–100 μm, capture signals from multiple cells within a single location, preventing single-cell resolution [24]. This limitation is particularly problematic for studying immune cell populations, which require high-resolution mapping for accurate characterization [17].

While sequencing-based ST methods have the potential to capture whole-transcriptome data, their effectiveness can also be constrained by probe capture efficiency, limiting the number of detectable transcripts. Consequently, the sequencing depth and coverage remain significantly lower than those of bulk RNA sequencing, making it challenging to detect genes with low expression [17,24].

Moreover, adequate preparation of samples for spatial analysis to maximize resolution involves a laborious process comprising multiple steps including tissue slicing, dissociation, and the incorporation of various barcodes and indexes [25]. Optimization procedures, such as adjusting tissue permeabilization conditions, may also be required to achieve optimal results. This intricate preparation process significantly increases experimental complexity and costs, making ST several times more expensive than bulk RNA sequencing with current technologies [26].

Resolution requirements may vary substantially across different applications in GU cancers, thus requiring a balance between analytical precision and sample throughput capabilities [26]. High-resolution ST platforms can generate single-cell or subcellular resolutions, enabling detailed characterizations of interactions within the tumor immune microenvironment; however, to maximize resolution, these approaches often require reduced sample throughput and increased computational power. Lower-resolution platforms may be more suitable for applications that require greater throughput capabilities, simplified data analysis, or for which broader spatial context is appropriate [26].

Spatial noise contributes to additional difficulty in correlating gene expression with subcellular locales [26]. This noise is primarily caused by mRNA diffusion during sample processing, which includes cryosectioning artifacts, variations in tissue permeabilization, and molecular drift in the process of RNA capture [27]. Classical computational methods including imputation-based approaches like Markov Affinity-based Graph Imputation of Cells, scImpute, and Single-cell Analyses Via Expression Recovery usually fail to correct for noise while often introducing false-positive signals that in turn further convolute spatial distributions of gene expressions [26]. As a countermeasure, SpotGF, an optimal transport-based gene filtering algorithm, specifically targets the removal of transcripts with high diffusion potential while preserving the in situ expression signal of the gene [26]. SpotGF, unlike other denoising algorithms that change the raw expression data from ST work, enhances the results of downstream analysis on cell clustering, cell type annotation, and lists of differentially expressed genes by correction for invalid genes contaminated through transcript diffusion [27]. This correction greatly augments the functional resolution of ST datasets, thereby counteracting the effects of spatial noise and enhancing the spatial precision of gene expression mapping [26]. SpotGF appears to be a viable computational strategy to counteract spatial resolution limits because of its ability to reject background noise and draw out true spatially distinct signals [27].

Newer methods of ST involving spatial barcode-based techniques, such as Slide-seq and High-Definition Spatial Transcriptomics (HDST), have achieved substantial resolution improvements, with HDST reporting a uniquely precise 2 µm resolution [27]. These improvements allow for mapping the gene expression at near single-cell resolution, a step crucial to assessing tumor heterogeneity and microenvironment interactions [28]. Nonetheless, some challenges in the spatiotemporal flow arising from limited RNA capture efficiencies and spatial misalignment still hinder present-day ST platforms from properly resolving complex tissue structures [27]. In this context, emerging technologies such as Deterministic Barcoding in Tissue for Spatial Omics Sequencing and Spatio-Temporal Enhanced Resolution Omics-sequencing (stereoseq) seek to facilitate spatial resolution enhancement with transcriptome-wide coverage [27]. The integration of these high-resolution ST methods with computational strategies based on Bayesian modeling and machine learning-based imputation may further refine spatial gene expression mapping and enhance accuracy in tissue segmentation applied to ST datasets [28].

Selecting the most appropriate ST platform depends on multiple factors including resolution requirements, tissue type, and experimental objectives. Each platform offers distinct advantages and limitations that must be carefully evaluated for specific research applications in genitourinary cancers (Table 2) [19].

#### 2.2.3. Computational Tools and Data Analysis

Analysis of spatial transcriptomic data consists of multiple downstream processes including conversion of sequencing data into gene expression matrices, dimensional reduction clustering, deconvolution, spatial trajectory inference, and cell–cell communication analysis [29]. Comprehensive analysis tools have been developed to standardize and unify data. However, most bioinformatics tools developed to date have limited capability to handle data with high complexity and dimensionality, particularly when integrating transcriptomic data with clinical and imaging data [29]. Ongoing contributions to spatial bioinformatics tools aimed at resolving these struggles include the STUtility and BayesSpace algorithms [30]. However, few pipelines specific to GU malignancies have been developed to date [25].

Computation has played a vital role in overcoming the inherent challenges of ST to allow for accurate characterization of spatial gene expression patterns [30]. Sparse gene expression data and signal dropout due to low RNA capture efficiency remain among the most significant challenges in ST, requiring computational approaches such as imputation techniques to recover missing expression patterns [30]. For example, Denoising and Imputing ST and BayesSpace train spatially aware smoothing functions to improve expression estimates using a random walk formulation while convolutional neural networks (CNNS) take a semi-supervised approach to tissue-specific joint embedding of spatial transcriptomic data and histological imaging to infer missing expression values at higher resolution [25]. Similarly, significant effort has been dedicated to developing normalization methods that enhance comparability between regions and across tissue sections, reducing technical variability while preserving the spatial distinctness of gene expression domains [30]. Another important computational advance entails integration of other datasets across different modalities, such as correlating ST data with scRNA-seq and histopathological imaging, allowing for more precise assignment of cell-types and identification of spatial domains. Each of these computational approaches is highly relevant for GU tumors, where appropriately tracing tumor-immune interactions, cellular heterogeneity, and therapeutic response signatures will allow for improved precision oncology approaches [25].

Beyond foundational computational approaches, advanced Bayesian modeling frameworks are emerging as particularly powerful tools for ST analyses, especially for overcoming challenges associated with spatial dependencies and gene co-expression networks [31]. Traditional spatial clustering techniques often go through dimensional reduction steps that affect the natural dependency structures existing between genes, limiting the accuracy of spatial domain segmentation. A joint Bayesian estimation (JOBS) framework was proposed to estimate gene co-expression and spatial dependencies in spatially resolved transcriptomic datasets [31]. In contrast to current methods which treat genes individually or work with preassigned spatial kernels, JOBS establishes a joint posterior distribution over the gene and spatial covariance matrices to better infer the spatial gene expression patterns [31]. This approach enhances the accuracy of spatial clustering and provides insights toward new cell types based on gene co-expression patterns. An additional advantage of JOBS is its ability to assess various independent tissue samples, which, through the Bayesian hierarchical model, enhances statistical power while accommodating spatial variability that is intrinsic to the unique samples [31]. Through simultaneously estimating gene expression patterns and spatial correlations, it provides a computational framework for advancing ST applications in GU cancer, where accurate tissue segmentation and molecular characterization are key for understanding tumor microenvironments and therapeutic opportunities [31].

A major hurdle in spatial clustering is balancing both resolution and segmentation sensitivity. Over-smoothing of spatial gene expression patterns commonly sacrifices fine-scale spatial variation, while over-fragmentation can create artificial boundaries that do not correspond to genuine biological structures [31]. A comprehensive benchmark analysis of spatial domain identification methods across multiple ST platforms provided a systematic framework for selecting optimal clustering approaches based on dataset characteristics [31]. The study evaluated methods across three key ST properties: differential gene expression profiles, spatial resolution, and gene coverage, while assessing computational robustness and scalability [31]. The results demonstrated that no single method performs universally across all datasets, with optimal performance being dataset-dependent. The study analysis also revealed challenges in processing large-scale datasets for which the authors propose a ‘divide and conquer’ strategy combining complementary analytical tools [31].

Deconvolution represents a critical computational challenge in ST, as individual capture spots often contain transcripts from multiple cell types, resulting in mixed signals that complicate accurate cell type identification [32]. A comprehensive benchmarking study of deconvolution methods was conducted and provides a systematic evaluation of deconvolution approaches, offering insights into their performance across various ST platforms and tissue types [32]. Deconvolution methods are broadly classified into reference-based and reference-free approaches. Reference-based approaches such as Robust Cell Type Decomposition, cell2location, and SPOTlight deconvolve a spatial spot based on its inferred cellular composition by referring to scRNA-seq data [32]. While these approaches generally achieve higher accuracy, they require high-quality, well-matched scRNA-seq reference datasets that may not be available [32]. Conversely, reference-free methods such as STdeconvolve and Conditional Autoregressive Deconvolution characterize cellular composition without external references, instead relying on spatial gene expression patterns to identify distinct cell populations [32]. While these methods are more flexible, they struggle with complex tissue architectures where gene expression profiles of distinct cell types overlap significantly [32]. Benchmarking analysis demonstrated that hybrid approaches, particularly Bayesian and graph-based models incorporating spatial transcriptomic data and biological priors, outperform purely data-driven methods in predominantly heterogeneous tissues such as tumors [32]. Importantly, the study provides practical guidelines for selecting appropriate deconvolution methods based on dataset characteristics, including spatial resolution, sequencing depth, and tumor complexity [32]. Deconvolution tools are critical for distinguishing malignant, stromal, and immune cell populations within the tumor microenvironment [32]. This capability enables precise characterization of cellular composition, ultimately enhancing ST’s utility for understanding disease progression, treatment response, and identification of novel therapeutic targets [32].

## 3. Current Clinical Insights of Spatial Transcriptomics in Genitourinary Cancers

### 3.1. Intra-Tumor and Inter-Tumor Heterogeneity Mapping

**Figure 2:**
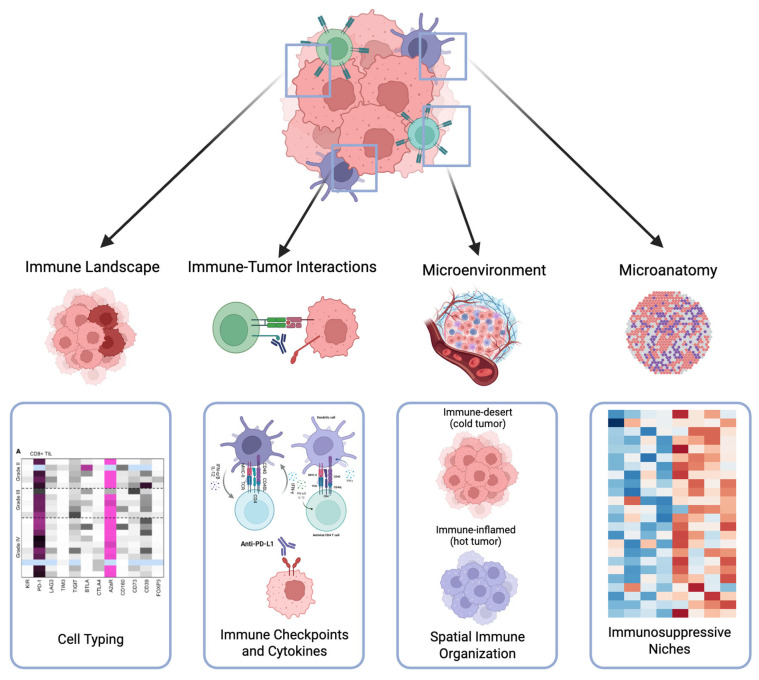
Spatial characterization of the tumor immune microenvironment in GU cancers. This schematic illustrates key components of the tumor immune ecosystem whose spatial organization is essential for understanding cancer pathogenesis and therapeutic responses. The framework encompasses immune landscape profiling, immune-tumor interactions including checkpoint pathways, microenvironmental factors governing immune cell infiltration patterns (immune-excluded versus immune-inflamed phenotypes), and immunosuppressive niches.

Spatial whole transcriptome profiling of primary tumors in metastatic urogenital cancers reveals profound tumor complexity characterized by extensive intra-tumoral heterogeneity (Figure 2) [33]. Renal cancers exemplify this complexity, encompassing diverse histopathological subtypes with distinct clinical characteristics, molecular profiles, and immunophenotypes [34]. Clear cell renal cell carcinoma (ccRCC), the most common histopathological subtype, demonstrates significant intra-tumoral heterogeneity and complex immune microenvironment dynamics that influence treatment response [35]. The success of immune checkpoint inhibition in metastatic RCC has highlighted the importance of understanding tumor-immune interactions, while the identification of distinct immunophenotypes has revealed potential therapeutic vulnerabilities. Recent molecular characterization efforts have identified key pathways involved in disease progression, including metabolic reprogramming through VHL/HIF pathway alterations and dysregulation of chromatin remodeling genes. Ongoing efforts aim to better characterize and identify novel therapeutic targets [36,37].

Similarly, urothelial carcinoma exhibits a diverse range of histopathologic characteristics and molecular changes, underscoring its morphological and genomic complexity [38]. This cancer is marked by a high frequency of somatic mutations, accompanied by significant genomic and transcriptional variability, which aligns with its broad spectrum of histological appearances and clinical presentations [39]. The expanded role of immunotherapy in the neoadjuvant setting in urothelial cancer has shown promise largely due to research highlighting favorable response rates of neoadjuvant immune checkpoint inhibitors in muscle invasive bladder cancer [40]. Immunohistochemistry for PD-L1, genomic alterations (i.e., tumor mutational burden), and molecular subtyping have shown potential as biomarkers for response in localized and advanced urothelial carcinoma [41,42,43].

Studies of muscle-invasive bladder cancer have identified specific mutations associated with reduced PD-L1 expression [44]. Results from these analyses demonstrated that mutations in tumor suppressor genes can disrupt immune evasion mechanisms by suppressing PD-L1 expression, thereby reducing the efficacy of PD-L1-targeted therapies [44]. Notably, mutations in TP53, commonly altered in muscle-invasive bladder cancer, were associated with decreased expression of immune-related genes including PD-L1 [45]. These findings suggest that loss of TP53 function may result in reduced therapeutic responsiveness due to impaired PD-L1 upregulation in response to immune system signals [45]. Multi-omics analyses have further revealed how mutations in other key genes, including those in the PI3K/AKT and MAPK pathways, regulate immune checkpoint expression [45]. These pathways, which are frequently dysregulated in cancer, can alter the tumor microenvironment by affecting immune cell infiltration and overall immune responses [45]. Pathway alterations result in PD-L1 downregulation, potentially compromising the efficacy of PD-L1-targeted immunotherapies [45].

Additional studies have highlighted the critical role of MTAP, CDKN2A, and CDKN2B in determining immune composition and anti-PD-L1 immunotherapeutic outcomes in muscle-invasive bladder cancer [44]. MTAP, an enzyme involved in adenine and methionine salvage pathways, is frequently co-deleted with the surrounding CDKN2A/CDKN2B locus in bladder cancer [44]. The tumor suppressors CDKN2A and CDKN2B encode negative regulators of cell cycle progression, and their loss promotes uncontrolled proliferation. MTAP deletion contributes to immune evasion through metabolite accumulation that suppresses immune cell activation and reduces PD-L1 expression [45]. This decreased PD-L1 expression enhances tumor immune evasion capabilities and impairs response to immune checkpoint blockade therapies [45].

In non-muscle-invasive bladder cancer (NMIBC), molecular tumor characteristics have been associated with pembrolizumab response patterns. BCG-resistant tumors characterized by inflamed PanCK+ tumor regions and infiltrated stromal areas demonstrate superior responses to intravenous pembrolizumab [46]. Conversely, immune-cold or non-inflamed tumors show inferior responses to intravenous pembrolizumab monotherapy, but exhibit the most favorable outcomes when treated with combination BCG and pembrolizumab therapy [46].

Recent advances in high-resolution molecular profiling have provided insights into prostate cancer heterogeneity and therapeutic resistance mechanisms. Using scRNA-seq and ST, researchers comprehensively analyzed prostate cancer tissues from 120 patients at single-cell resolution while preserving spatial context [47]. These analyses identified a distinct epithelial cell subtype, termed club-like cells, that functions as an intermediary between prostate tissue and the immune system [47]. These cells localized to regions with diminished androgen signaling, a pathway critical for prostate cell proliferation [47]. Furthermore, characterization of genes involved in androgen signaling and testosterone metabolism has elucidated potential mechanisms underlying castration resistance in prostate cancer [48].

Another study performed whole transcriptome profiling of primary tumors from patients with metastatic prostate cancer, identifying various cell types including cancer-associated fibroblasts (CAFs), endothelial cells, and immune cells, while mapping their spatial distribution within the tumor microenvironment [33]. The analysis revealed regions with an immune-excluded phenotype, where immune cells accumulate at the tumor periphery but fail to infiltrate the tumor core, potentially facilitating immune evasion [33]. Furthermore, the analysis characterized CAF-enriched stromal areas that secrete immunosuppressive factors such as TGF-β and remodel the extracellular matrix to promote tumor dissemination [33].

Other studies have demonstrated that the tumor microenvironment exhibits spatial heterogeneity rather than uniform immunosuppression, with distinct “cold” (immune-suppressed) and “hot” (immune-active) zones coexisting within individual prostate tumors [49]. This tumor heterogeneity underscores the critical value of personalized molecular profiling approaches. Additionally, spatial gene expression analysis revealed significant differences between tumor core and periphery regions, providing important insights into prostate cancer development and progression mechanisms [49]. The stromal compartment emerged as a key driver of cancer initiation and progression through spatial analysis [49]. From a clinical perspective, detailed examination of normal and inflamed stromal regions, as well as normal and prostatic intraepithelial neoplasia glands, may enable detection of early carcinogenic processes [50]. The continued identification of novel biomarkers and targeting of specific genetic mutations represent promising approaches for developing precision therapies in advanced prostate cancer [50,51].

In penile cancer, recent studies have revealed that HPV-positive and HPV-negative penile squamous cell carcinomas display biologically distinct pathways [52]. ScRNA-seq and T-cell receptor sequencing have provided a comprehensive atlas of the cellular architecture of penile squamous cell carcinoma, demonstrating that patients with aberrant p53 (TP53LOF) exhibit significantly worse outcomes than patients with wild-type p53 expression, regardless of HPV status [52]. TP53LOF tumors display epithelial-to-mesenchymal transition (EMT), immune exclusion, and angiogenesis irrespective of HPV status [52]. Comprehensive scRNA-seq analysis revealed that HPV-positive TP53 wild-type patients exhibit E2F signaling activation, increased proliferation, and terminal epithelial maturation [52]. This was characterized by a high prevalence of cornified and superficial ridge cells that strongly express novel antibody-drug conjugate targets, including NECTIN4 and TROP2 [52]. Conversely, TP53LOF patients demonstrated an aggressive phenotype characterized by cancer cells that failed to undergo complete epithelial maturation and exhibited strong partial EMT activation [52]. The observed correlation between partial EMT, capillary tip cells, and cancer-associated fibroblasts suggests potential tumor-stromal interactions that may promote metastasis [52].

### 3.2. Early Detection and Biomarker Discovery

#### 3.2.1. Role of Fibroblasts and Stromal Cells

CAFs modulate tumor and immune cell functions through cytokine secretion, extracellular vesicle release, direct cellular interactions, and extracellular matrix remodeling [53]. ScRNA-seq of bladder cancer has identified two functionally distinct CAF subtypes. PDGFRα+ inflammatory CAFs (iCAFs) highly express chemokines, cytokines, and genes associated with extracellular matrix degradation, cell migration, and angiogenesis [53]. These iCAFs represent the predominant source of CXCL12, which plays a critical role in tumor-associated macrophage recruitment, with elevated CXCL12 levels correlating with poor prognosis and increased macrophage infiltration. In contrast, RGS5+ myofibroblastic CAFs demonstrate enriched expression of genes involved in extracellular matrix organization and focal adhesion pathways [53].

Furthermore, scRNA-seq and ST have identified a novel CAF subset expressing PDGFRα+ITGA11+, which plays a critical role in early-stage bladder cancer progression by promoting lymphovascular invasion and lymph node metastasis [53]. Additional studies have characterized a distinct fibroblast population termed the S3 cluster, which is predominantly found in bladder cancer and associated with poor prognosis [54]. Tumors enriched with S3 fibroblasts demonstrate increased blood vessel density, suggesting a key role in angiogenesis [54]. The NOTCH1-JAG2 signaling pathway mediates interactions between fibroblasts and endothelial cells, while YAP1, a transcription factor, has been identified as a potential regulator of the S3 fibroblast phenotype, providing molecular insights into their unique functional properties [54].

In prostate cancer, spatial analysis has revealed stromal heterogeneity with distinct functional consequences [21]. Regions densely populated with CAFs promote tumor growth, invasion, and treatment resistance, while areas predominantly composed of normal stromal cells may exert tumor-suppressive effects [21]. This stromal heterogeneity highlights the importance of considering regional differences when developing therapeutic approaches [21]. Additionally, prostate cancer studies have identified specific metastatic niches within tumors where aggressive cancer cells co-localize with immunosuppressive immune cells, including myeloid-derived suppressor cells (MDSCs) and regulatory T-cells (Tregs), likely contributing to treatment resistance [33].

#### 3.2.2. Role of Androgen Receptors in Prostate Cancer Progression

**Figure 3:**
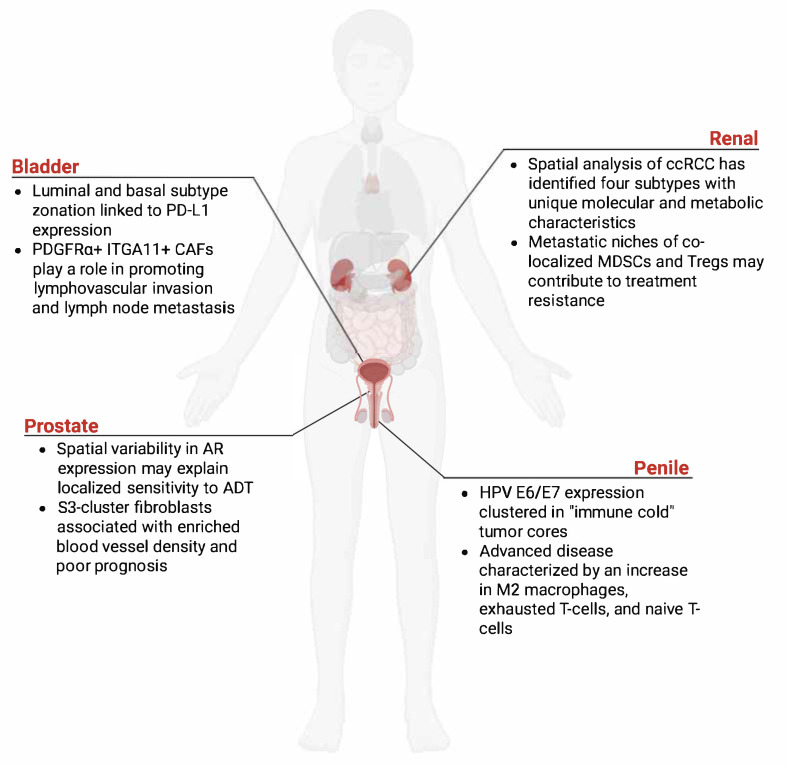
ST application in GU research. ST use has helped identify cellular environment and construct GU spatial atlas. In both bladder cancer and prostate cancer, tumor microenvironment, growth factor and receptors play a role in cancer cell progression. Other applications of ST include identifying metastatic niches in Renal cancer and HPV specific expression in “immune cold” penile tumor cores associated with M2 macrophages exhausted T-cells [21,41,52,53,54,56,57]. CAFs: cancer-associated fibroblasts; AR: Androgen Receptor; ADT: Androgen Deprivation therapy; ccRCC: clear cell Renal Cell Carcinoma; MDSCs: myeloid-derived suppressor cells; Tregs: regulatory T-cells; HPV: Human Papillomavirus.

The androgen receptor (AR) signaling pathway is central to prostate cancer progression and treatment, particularly in the context of androgen deprivation therapy [21,48]. Under normal physiological conditions, androgens bind to their receptors to facilitate essential cellular functions including growth and division [21]. In prostate cancer, this pathway becomes dysregulated, driving uncontrolled tumor cell proliferation (Figure 3) [21].

Spatial analysis has revealed significant heterogeneity in AR activity within individual tumors [21]. While certain tumor regions exhibit high AR activity, other areas demonstrate reduced AR signaling or enrichment in AR-independent pathways [21,33,48,55]. This spatial variation suggests that some tumor regions remain sensitive to androgen deprivation therapy, while others may drive resistance through alternative growth pathways [55]. This intra-tumoral heterogeneity underscores the challenges of uniform treatment approaches and highlights the need for spatially informed therapeutic strategies [21].

Mutations within AR, particularly in the ligand-binding domain, can alter receptor conformation and activity, contributing to disease progression [55]. The T887A mutation enhances receptor sensitivity to antiandrogens, leading to ligand-independent activation and continued tumor growth despite appropriate treatment [55]. Similarly, the W741C mutation complicates treatment by converting antiandrogens into receptor agonists. Understanding these AR mechanisms is crucial for developing targeted therapies and improving patient outcomes in prostate cancer [47].

Additionally, club-like cells have been shown to secrete factors characteristic of the senescence-associated secretory phenotype, which influence neighboring cell behavior [47]. The presence of club-like cells correlates with increased activity of polymorphonuclear MDSCs, immune cells that suppress anti-tumor immune responses [47].

#### 3.2.3. Metabolic Heterogeneity in Cancer Progression

Spatial transcriptomic studies have revealed marked metabolic heterogeneity within prostate tumors, with distinct metabolic profiles driven by regional variations in gene expression [21]. Some tumor regions demonstrate heightened lipid metabolic activity, reflecting prostate cancer’s dependence on lipids for energy production and cell membrane synthesis [21,49]. Other areas exhibit elevated activity in both glycolytic and oxidative phosphorylation pathways, indicating metabolic flexibility in response to local microenvironmental conditions such as oxygen availability and nutrient supply [21]. This metabolic heterogeneity suggests that targeting single metabolic pathways may be insufficient, as different tumor regions rely on diverse energy generation mechanisms [21]. Spatial analysis has further identified regions enriched in oxidative phosphorylation and fatty acid metabolism genes, while other areas demonstrate dependence on glycolytic pathways [21].

Additionally, hypoxic regions characterized by low oxygen tension exhibit upregulation of hypoxia-inducible factor pathways and angiogenic genes [58]. These hypoxic areas may contribute to treatment resistance and tumor aggressiveness by creating poorly vascularized microenvironments [58].

The complex interplay between the tumor microenvironment and cancer cells demonstrates that metabolic adaptations are driven not only by intrinsic tumor characteristics but also by surrounding environmental factors, highlighting the importance of considering spatial context in therapeutic targeting strategies [59].

### 3.3. Drug Resistance Mechanisms

#### Spatial Profiling of Resistance-Associated Pathways

Spatial transcriptomic analysis of 100 ccRCC patients identified four distinct molecular subtypes with unique resistance mechanisms [57]. The dedifferentiated ccRCC subtype demonstrated distinctive molecular characteristics accompanied by metabolic reprogramming involving alterations in TCA cycle, glycolysis, and lipid metabolism pathways [57]. These metabolic changes may contribute to drug resistance by enabling tumor adaptation to therapeutic interventions [57]. Integrated single-nucleus RNA sequencing (snRNA-seq) and single-nucleus assay for transposase-accessible chromatin using sequencing (snATAC-seq) analysis of human ccRCC specimens identified twenty tumor-specific markers [57]. A notable finding was the association of ceruloplasmin (CP) with ccRCC progression [57]. CP functions primarily in copper transport, ferroxidase activity, angiogenesis, and oxidative stress regulation [57]. Bulk and scRNA-seq revealed CP overexpression in ccRCC compared to normal adjacent tissue and other RCC subtypes [57]. CP expression correlates with higher tumor grades and poor prognosis in ccRCC studies. Functional studies demonstrated that CP knockdown reduced the invasive capacity of RCC cells, suggesting its potential role in therapeutic resistance [57]. Additionally, chromatin accessibility analysis revealed that BAP1 mutations result in global decreased chromatin accessibility, while PBRM1 mutations predominantly increase accessibility [57].

## 4. Challenges and Future Frontiers in Spatial Transcriptomics for Genitourinary Cancers

### 4.1. Challenges Facing ST for GU Cancers

The integration of ST into clinical practice represents an emerging area of advancement in cancer diagnostics; however, rigorous validation is still needed to bridge the gap between research findings and routine clinical practice [14,28,36,60]. While classical histopathology has served as the foundation of cancer diagnosis for nearly two centuries, providing essential insights into tissue morphology and cellular architecture, NGS and single-cell profiling have revolutionized our understanding of tumor heterogeneity and microenvironmental interactions [61].

Clinical adoption of ST platforms requires addressing several critical challenges, including the need for prospective clinical validation studies, development of bioinformatics tools for large-scale data integration, and establishment of high-throughput standardized workflows [62]. The clinical utility of ST depends fundamentally on the reproducibility and reliability of analytical results, representing the primary limitation for clinical implementation [63]. Throughput and scalability considerations are particularly important for biomarker validation studies and clinical deployment [25]. Robust molecular biomarker development requires analysis of large patient cohorts to account for disease heterogeneity and ensure adequate statistical power, necessitating evaluation of per-sample processing costs, batch processing capabilities, and sample preparation timelines [64]. Clinical integration introduces additional constraints including turnaround time from sample collection to result delivery, workflow integration with existing diagnostic processes, and quality control measures [65]. Platforms offering automated sample processing and standardized analysis may achieve broader adoption despite higher infrastructure requirements [65].

Cost and scalability remain significant barriers to widespread implementation of ST in GU oncology [66]. Current platforms require substantial financial investment and are primarily accessible to well-funded research institutions [66]. Clinical translation will require cost-effective and scalable technological solutions, potentially through assay miniaturization or workflow simplification without compromising analytical accuracy [66].

The lack of standardized protocols for ST applications in GU cancers presents another challenge [62]. Variability in tissue handling, data acquisition, and analysis pipelines across studies affect reproducibility and cross-study comparisons [62]. Establishing standardized guidelines and quality metrics specific to GU oncology will be essential for accelerating clinical translation [67].

### 4.2. Advanced Technologies for 3D Tumor Mapping

**Figure 4:**
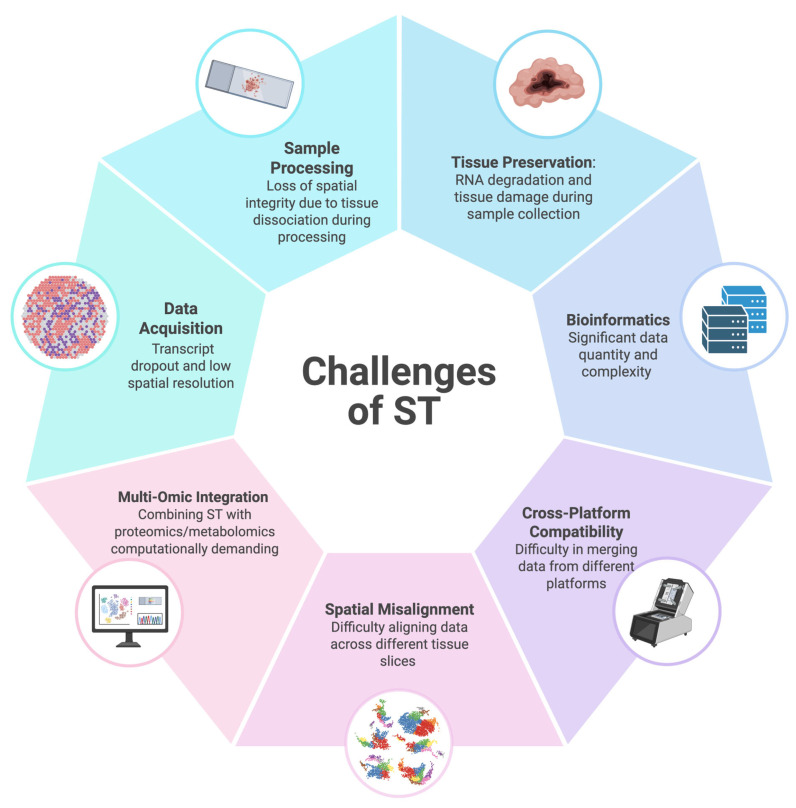
Key challenges in spatial transcriptomics (ST) including sample processing artifacts that compromise spatial integrity, tissue preservation issues leading to RNA degradation, bioinformatics complexity due to large dataset sizes, cross-platform compatibility, spatial misalignment difficulties across tissue sections, and computational demands of multi-omic data integration.

While ST is increasingly utilized in cancer research, it remains a sophisticated platform with significant technical and analytical challenges that limit its broad applicability (Figure 4) [26,39,68,69,70,71]. RNA degradation during tissue collection represents a primary concern, as it can lead to altered RNA sequencing profiles [72]. This limitation necessitates rapid RNA preservation through immediate cryopreservation and controlled freezing protocols [72]. Additionally, the relatively low spatial resolution of current ST platforms, which may capture transcripts from multiple cells simultaneously, can compromise single-cell resolution analysis [68]. Furthermore, integrating data from different platforms presents computational challenges and cross-platform compatibility issues [69].

Despite these limitations, ST has advanced our understanding of GU cancer architecture compared to traditional two-dimensional approaches that inadequately captured spatial tumor organization [28,70]. Three-dimensional ST enables visualization of gene expression patterns across complex tumor structures, providing valuable insights into the dynamic interactions between tumor cells, stromal components, and immune infiltrates [17,28]. This technology is particularly valuable for mapping tumor cell development and microenvironmental interactions during cancer progression [70]. For instance, spatial mapping of metastatic prostate cancer has revealed gene expression patterns associated with organotropism in bone and liver metastases, identifying potential therapeutic targets [73].

Sequential tissue sampling combined with spatial analysis enables temporal mapping of tumor evolution, facilitating studies of progression and treatment resistance over time [28]. Longitudinal prostate cancer studies have characterized AR signaling changes during androgen deprivation therapy, elucidating key resistance mechanisms [55].

The integration of three-dimensional ST with diagnostic workflows enables patient stratification based on unique molecular and spatial tumor profiles, guiding personalized treatment approaches [17,23,28]. This precision medicine strategy ensures patients receive therapies tailored to the specific molecular and spatial characteristics of their individual tumors [74].

This figure summarizes the key challenges of ST, including lack of tissue preservation due to RNA degradation during collection [26], loss of spatial integrity during sample processing, low spatial resolution affecting data acquisition [68], computational demands caused by combining data from different sources [69], and spatial misalignment when trying to align highly complex data across different slices [71].

### 4.3. Multi-Omics and AI Integration

Given the extensive molecular heterogeneity of cancer cells, integrating ST with multi-omics datasets enhances our understanding of tumor biology [62]. Studies have combined ST with scRNA-seq to elucidate relationships between spatial gene expression patterns and mutational landscapes or epigenetic modifications in bladder and prostate cancers [75].

The convergence of artificial intelligence (AI) and multi-omics data is advancing new approaches in ST for GU cancers, enabling comprehensive characterization of tumor microenvironments and disease progression [62,76]. Deep learning approaches that integrate ST with complementary modalities including histology, scRNA-seq, chromatin imaging, and proteomics are advancing spatial resolution capabilities, cellular deconvolution, and biomarker identification [76].

Deep learning models for ST integration include graph neural networks (GNNs), variational autoencoders (VAEs), and contrastive learning techniques [76]. GNNs leverage spatial information to refine transcriptomic clustering and cellular neighborhood inference, while VAEs enable imputation of missing gene expression values, enhancing analytical robustness [76]. Integration with histological and chromatin imaging provides morphological and nuclear context to complement spatial transcriptomic data [76]. Neuropathology Spatial Transcriptomic Analysis (NePSTA) exemplifies the potential of combining ST with GNNs for comprehensive molecular and histopathological diagnostics, achieving highly accurate automated tumor subtyping [76]. This graph-structured deep learning approach integrates transcriptomic gene expression profiles with tissue spatial architecture to enhance prediction of tumor histology and molecular subtypes [76]. Validation across multiple cohorts demonstrated 89.3% accuracy in predicting tissue histology and methylation-based subclasses [76]. Notably, NePSTA can generate immunohistochemistry profiles through ST and infer genomic variations from minimal tissue samples, addressing limitations in cases with insufficient DNA for conventional molecular diagnostics [77]. The NePSTA framework significantly enhances biomarker discovery by integrating inferred immunohistochemistry with ST [77]. Protein abundance prediction with spatial resolution through Bayesian inference methodologies serves as a proxy for conventional immunohistochemical staining [77]. This enables direct examination of spatial distribution patterns for key markers such as Ki67, GFAP, and NeuN from transcriptomic data, transforming tumor classification and microenvironmental interaction analysis [77].

CNNs and transformer-based architectures extract features from histological images in conjunction with corresponding gene expression data, enhancing identification of spatial domains within tumor tissues and enabling refined mapping of heterogeneous cell populations and their interactions with tumor-associated immune cells [76]. Chromatin imaging integration has facilitated identification of joint molecular and structural biomarkers, providing insights into chromatin accessibility and transcriptional regulation during cancer progression [76].

Several challenges must be addressed as AI-driven approaches continue to proliferate in order to handle increasingly complex ST datasets. High-throughput computational models are needed to process thousands of spatially resolved data points while maintaining model interpretability. Future research should focus on strengthening deep learning models for enhanced spatial resolution, improving cross-modal alignment, and generating clinically actionable insights from integrated multi-omics datasets. AI-facilitated multimodal integration positions ST to contribute to advances in GU cancer understanding for precision oncology strategies [76].

### 4.4. Advancing Targeted Therapy in GU Oncology Through ST

Clinical trials have demonstrated the utility of ST in predicting immunotherapy responses. Studies in NMIBC revealed that immune-cold tumors showed limited response to intravenous pembrolizumab monotherapy but exhibited favorable outcomes when treated with combination BCG and pembrolizumab therapy [46]. This spatial characterization enables stratification of patients for optimal immunotherapy approaches based on their unique tumor immune landscape [46].

Understanding immune-tumor interactions through spatial mapping facilitates the development of targeted immunotherapies, including personalized cancer vaccines designed against spatially defined tumor antigens [53]. Knowledge of immune cell exclusion patterns and suppression mechanisms in specific tumor regions can enable strategies to reprogram the immune microenvironment and overcome resistance [53].

ST has proven valuable in predicting responses to kinase inhibitors, a major class of targeted therapeutics [74]. RNA velocity analysis has identified opportunities to target drugs that can shift tumor cells from invasive, proliferative phenotypes to more differentiated states, potentially reversing tumor cell plasticity and reducing metastatic capacity. CDK inhibitors, including Alvocidib, have demonstrated the ability to alter transcriptional states and inhibit EMT signaling, a key driver of cancer invasion and metastasis [74,78]. Similar findings in renal cell carcinoma have validated the role of CDK inhibitors in promoting tumor differentiation and suppressing growth [78].

The clinical implementation of ST faces several key challenges, including the need for standardized protocols across laboratories to ensure reproducible results between institutions [79]. Additional barriers include the high costs of current technologies and the development of user-friendly analysis platforms accessible to clinicians without specialized bioinformatics expertise [79].

### 4.5. Standardized ST Data Sharing

As spatial transcriptomic technologies advance, standardized data sharing has become essential for ensuring transparency, reproducibility, and interoperability across research platforms [80]. Unlike conventional sequencing approaches, ST generates complex datasets that integrate imaging, sequencing, and metadata components, significantly complicating standardization efforts [80].

Fluorescence-based microscopy spatial transcriptomic techniques produce large-scale datasets, raising critical questions about optimal data sharing practices [80]. Raw images enable complete reanalysis and validation of findings but require substantial storage capacity and specialized technical expertise for processing [80]. Pre-processed images offer greater accessibility but may introduce analytical biases based on author-selected processing parameters [80]. Community guidelines recommend adopting the Open Microscopy Environment (OME)-TIFF format to ensure cross-platform compatibility [80]. Emerging formats such as OME-NGFF are being developed specifically to address scalability challenges, enabling cloud-based storage and integration of multimodal spatial datasets [80].

Segmentation defines transcript localization in spatial transcriptomic studies and is crucial for downstream analysis [80]. Segmentation masks annotate image regions corresponding to cells or tissue structures and are widely utilized in machine learning applications, including CNNs [80]. Sharing segmentation information alongside raw images enhances reproducibility and provides standardized comparison frameworks [80]. Segmentation data should be provided in both image mask and coordinate-based formats to ensure compatibility with diverse analytical tools. Segmented objects should include unique identifiers to facilitate downstream integration processes [80].

Many spatial transcriptomic platforms utilize NGS to quantify gene expression in spatially indexed regions [80]. Raw sequencing data should be deposited in established repositories including the NCBI Gene Expression Omnibus, European Genome-Phenome Archive, or ArrayExpress with standardized metadata annotation, ensuring compliance with FAIR data principles [80]. Comprehensive spatial barcoding documentation is particularly critical for technologies employing indexed transcript capture rather than direct imaging detection [80].

Expression matrices represent the primary analytical output of ST, capturing gene expression levels in spatially resolved units [80]. Transparent sharing of unprocessed count matrices with detailed data processing workflow descriptions enables independent reanalysis and validation [80]. Essential metadata including spatial coordinates, tissue annotations, and experimental conditions must conform to established geospatial data standards [80]. Standardized frameworks such as SpatialFeatureExperiment in R v4.3.0+ and SpatialData libraries in Python v3.10+ are advancing standardized metadata structures to enable platform interoperability [80].

## 5. Conclusions

ST has significantly advanced GU cancer research by enabling spatially resolved gene expression analysis within tissue architecture [25,28,32,62,72,74]. This technology bridges the gap between molecular biology and histological context, providing insights into tumor cell interactions with immune cells and surrounding stroma [28,74]. Studies across prostate cancer, bladder cancer, and renal cell carcinoma have revealed extensive tumor heterogeneity with spatial patterns that correlate with clinical outcomes, including drug resistance mechanisms and prognostic indicators [43,55,57,58,75,78]. Comprehensive profiling of immune landscapes and stromal components has illuminated tumor microenvironment dynamics, including the spatial organization of immunosuppressive niches and cancer-associated fibroblast roles in disease progression [74]. Furthermore, ST has advanced biomarker discovery by identifying spatially resolved molecular signatures undetectable through conventional bulk sequencing approaches [19,28,74,75,78]. These achievements collectively establish ST as a cornerstone of modern GU cancer research and an essential tool for precision oncology strategies [74,79,80].

Continued advancements in spatial transcriptomic technology may inform changes to cancer diagnosis, treatment, and management paradigms [61,72,74,77]. Emerging three-dimensional spatial transcriptomic technologies can capture both spatial organization and temporal cancer evolution, enabling detailed metastasis studies [61,72,74,77]. Three-dimensional architectural mapping of cancers may reveal critical progression pathways previously inaccessible to analysis. Integration of multi-omics approaches, including proteomics, metabolomics, and epigenomics with ST, will provide comprehensive tumor biology characterization underlying GU cancer pathogenesis and therapeutic resistance [44,45,61,72,74,77,80].

Artificial intelligence and machine learning will likely play a central role in analyzing the large datasets generated by ST, facilitating discovery of novel therapeutic targets and predictive biomarkers [76,77]. These computational tools can decode complex spatial patterns governing immune dynamics and tumor-stromal interactions, enabling breakthroughs in immunotherapy and personalized vaccine development [76,77]. Extension of ST to rare GU cancers, including testicular cancer and non-urothelial bladder malignancies, will address critical knowledge gaps and provide novel clinical intervention opportunities [81,82]. Realizing this vision will require continued innovation, interdisciplinary collaboration, and sustained efforts to bridge the research-to-clinical practice gap.

Substantial investment is needed to make spatial transcriptomic technologies more accessible and scalable [66]. Current high costs and technical complexity restrict utilization to specialized research centers, limiting broader impact [26,64,79]. Future clinical application of spatial transcriptomics for routine diagnostic and therapeutic decision-making in genitourinary cancers will require cost reduction, workflow standardization, and extensive validation studies to bridge the current gap between research applications and clinical implementation [29,63,80]. Academic-biotech partnerships may help accelerate translational development of clinical spatial platforms, while multidisciplinary collaborations could foster integration between spatial data, advanced imaging and complementary analytical modalities [44,45,76,77].

## Figures and Tables

**Table 1:** Comparison of commercially available ST platforms.

Platform	Company	Methodology	Throughput *	Processing Time *	Maximum Targets *	Resolution *	Sample Types
Xenium	10x Genomics	Padlock probe with rolling circle amplification	2–4 samples/run	2–3 days	5000 RNAs	Subcellular	FFPE, Fresh frozen
Visium	10x Genomics	Spatially barcoded spots for mRNA capture and sequencing	8–16 samples/run	2–3 days	All 3′ mRNA	55 μm (single-cell with HD)	FFPE, Fresh frozen; Larger tissue sections preferred
Stereo-seq	BGI Genomics/MGI	DNA nanoball arrays for RNA capture and sequencing	4–8 samples/run	2–3 days	All 3′ mRNA	500 nm	Fresh frozen preferred; FFPE possible
Seeker	Curio Bioscience	Spatially barcoded bead arrays	4–8 samples/run	2–3 days	All 3′ mRNA	10 μm	Fresh frozen
PhenoCycler	Akoya Biosciences	Cyclic immunofluorescence with barcoded antibodies for protein detection	2–4 samples/run	1–2 days	~100 proteins	Single cell	FFPE, Fresh frozen
COMET	Bio-Techne	Sequential immunofluorescence with repeated staining/imaging cycles in microfluidics	4–8 samples/run	1 day	40 proteins	Single cell	FFPE preferred
CellScape	Bruker/Canopy	Iterative fluorescent antibody staining/bleaching cycles in a microfluidic chip	4–8 samples/run	1 day	30 proteins	Single cell	FFPE on coverslips
CosMx	NanoString	Branched DNA probes with multiple readout sequences	1–4 samples/run	2–3 days	18,000+ RNAs	Subcellular	FFPE, Fresh frozen
MERSCOPE	Vizgen	Multiple probes per RNA with unique readout sequences	4–8 samples/run	1–2 days	1000 RNAs	Subcellular	FFPE, Fresh frozen
GeoMx	NanoString	UV-cleavable oligo tags on probes	1–4 samples/run	1–2 days	18,000+ RNAs or 570+ proteins	~10 μm	FFPE, Fresh frozen
MIBIscope	Ionpath	Metal-labeled antibodies detected by mass spectrometry	2–4 samples/run	1–2 days	40 proteins	290 nm–1 μm	FFPE, Fresh frozen

FFPE: formalin-fixed paraffin embedded; mRNA: messenger RNA; HD: high definition; RNA: ribonucleic acid; UV: ultraviolet; nm: nanometer; μm: micrometer. * Some details, such as exact throughput, processing times, targets, and resolution may vary based on experimental setups, sample preparation, and platform configurations.

**Table 2:** Platform selection recommendations based on experimental needs in GU cancers.

Experimental Need	Recommended Platforms
Whole transcriptome, bulk TME profiling	10X Visium Visium HD, Streoseq, GeoMx DSP
Single-cell resolution for tumor heterogeneirty	Visium HD, Stereoseq, Xenium, Merscope, CosMx
Sub-cellular resolution for cell–cell interactions	Stereoseq, Xenium, Merscope, CosMx
RNA + protein co-detection	GeoMx DSp, Merscope, CosMx
High-throughpt, cost-effective spatial profiling	10X Visium, Stereoseq
Best for small biopsies or targeted ROIs	GeoMx DSP
Best for degraded RNA (FFPE samples)	Xenium, Visium HD, GeoMx, DSP

## Abbreviations

The following abbreviations are used in this manuscript: ADT: Androgen Deprivation Therapy; AR: Androgen Receptor; CAFs: cancer-associated fibroblasts; ccRCC: clear cell Renal Cell Carcinoma; CNNs: Convolutional neural networks; CP: ceruloplasmin; DOAJ: Directory of open access journals; DSP: Digital Spatial Profiling; EMT: Epithelial-to-mesencymal transition; FFPE: formalin-fixed paraffin-embedded; FISH: Fluorescence in situ hybridization; GNNs: graph neural networks; GU: Genitourinary; HDST: High-Definition Spatial Transcriptomics; HPV: Human Papillomavirus; iCAFS: inflammatory CAFs; ISH: In situ hybridization; JOBS: joint Bayesian estimation; LCM: laser capture microdissection; LD: Linear dichroism; MDPI: Multidisciplinary Digital Publishing Institute; MDSCs: myeloid-derived suppressor cells; NEPSTA: Neuropathology Spatial Transcriptomic Analysis; NGS: next-generation sequencing; NMIBC: non-muscle-invasive bladder cancer; OME: Open Microscopy Environment; scRNA-seq: single-cell RNA sequencing; seqFISH: sequential fluorescence in situ hybridization; SMI: Spatial Molecular Imaging; ST: Spatial Transcriptomics; TLA: Three letter acronym; Tregs: regulatory T-cells; VAEs: variational autoencoders
